# The Sequence of Steps: A Key Concept Missing in Surgical Training—A Systematic Review and Recommendations to Include It

**DOI:** 10.3390/ijerph20021436

**Published:** 2023-01-12

**Authors:** Victor Galvez-Yanjari, Rene de la Fuente, Jorge Munoz-Gama, Marcos Sepúlveda

**Affiliations:** 1 Department of Computer Science, School of Engineering, Pontificia Universidad Católica de Chile, Santiago 8331150, Chile; 2 Division of Anesthesiology, School of Medicine, Pontificia Universidad Católica de Chile, Santiago 8330024, Chile

**Keywords:** sequence of steps, teaching, assessment, procedural skills

## Abstract

Background: Surgical procedures have an inherent feature, which is the sequence of steps. Moreover, studies have shown variability in surgeons’ performances, which is valuable to expose residents to different ways to perform a procedure. However, it is unclear how to include the sequence of steps in training programs. Methods: We conducted a systematic review, including studies reporting explicit teaching of a standard sequence of steps, where assessment considered adherence to a standard sequence, and where faculty or students at any level participated. We searched for articles on PubMed, EMBASE, CINAHL, Web of Science, and Google Scholar databases. Results: We selected nine articles that met the inclusion criteria. The main strategy to teach the sequence was to use videos to demonstrate the procedure. The simulation was the main strategy to assess the learning of the sequence of steps. Non-standardized scoring protocols and written tests with variable validity evidence were the instruments used to assess the learning, and were focused on adherence to a standard sequence and the omission of steps. Conclusions: Teaching and learning assessment of a standard sequence of steps is scarcely reported in procedural skills training literature. More research is needed to evaluate whether the new strategies to teach and assess the order of steps work. We recommend the use of Surgical Process Models and Surgical Data Science to incorporate the sequence of steps when teaching and assessing procedural skills.

## 1. Introduction

Surgeons receive training on a wide variety of skills, including procedural skills. Procedural skills are composed of affective, cognitive, and psychomotor domains [1], which are complex to develop because they are interdependent on each other [2,3]. In particular, the psychomotor domain includes the ability to perform skills that involve movements [4], and Dave’s taxonomy establishes that such domain is developed by imitating the skill until performing the procedure naturally, in a logical sequence [5].

Procedural variability refers to differences in how surgeons perform procedures [6,7]. These variations occur due to different surgeons’ backgrounds and individual preferences [8]. The variability is present when surgeons embrace challenging situations in the operating room, and even when procedures are practiced in simulation-based contexts, where external factors such as resources and patient variability do not exist [8]. Particularly, it has been shown that variability in the sequence of steps is the rule and not the exception [9,10,11,12]. Furthermore, instructors believe that dealing with procedural variability is a relevant aspect that residents need to learn [7], but it is difficult for residents to recognize what is a principle (i.e., a mandatory step) and what is an individual preference (i.e., options to perform a step) [6] in every different way to perform the procedure. Hence, the sequence of steps needs to be considered in training programs to improve residents’ learning [6,7,13].

Standardization of surgical procedures is key to making residents deal with procedural variability in training programs, which is positive to increase the surgical quality and the patient safety [14,15,16]. This process involves the standardization of the sequence of steps through the consideration of the different paths a surgical procedure have and the decision-making on what is relevant to include in the standard generated [10,17]. The standardization of the sequence of steps allows one to establish the competence that residents must acquire at the end of the training, which helps to design novel strategies to be used in competency-based training programs [18]. For this task, McKinley et al. (2008) [19] presented the essential competencies that residents should learn to perform surgical procedures, including procedural competence, which involves the sequence of steps. Moreover, it is known that surgical procedures can be represented as a set of sequentially ordered steps [10,20]. Therefore, we searched the literature for articles reporting the explicit incorporation of a standard sequence of steps in training programs, particularly into teaching and assessment strategies of procedural skills training, with the aim of determining current practices that consider the sequence of steps aspect of procedural skills. By “explicit”, we mean showing linked but separate steps as a sequence in a concrete way, e.g., through BPMN (Business Process Model and Notation) process models [17].

We conducted a systematic review to identify reported strategies considering the teaching and assessment of a standard sequence of steps in procedural skills training. Then, we present recommendations to include the sequence of steps into procedural skills teaching and assessment training strategies.

## 2. Methods

### 2.1. Research Questions

We seek to answer the following questions: (a) What strategies have been reported in instructional design studies to teach a standard sequence of steps in procedural skills training? Are these strategies effective? (b) What strategies, instruments, and outcomes have been reported to assess the learning of the sequence of steps in procedural skills training? Is there valid evidence for these instruments?

We considered as strategy any action performed to explicitly teach or assess the sequence of steps (e.g., showing in a printed flowchart the standard sequence or saying the steps aloud in the standard order) [21]. Moreover, we considered as an instrument any tool to assess the learning of the sequence of steps.

### 2.2. Protocol

To conduct this systematic review, we created a protocol following the Preferred Reporting Items for Systematic Reviews and Meta-Analyses (PRISMA) statement [22]. The protocol is presented as Supplementary Material.

### 2.3. Eligibility Criteria

We included original and full-text articles, published before 30 September 2019, in which participants were explicitly taught about a standard sequence of steps, or the assessment of learning considered whether participants’ performance adhered to a standard sequence of steps. Particularly, we included articles reporting that the standard sequence of steps was taught using any strategy to make it explicit, or the assessment considered whether students omitted steps, repeated steps, or performed steps in the wrong sequence. Moreover, we included studies where the participants were faculty or students at any level. We did not restrict articles’ inclusion to any specific specialty, study design, or training level (undergraduate, postgraduate, or staff).

We excluded articles that considered teaching or assessing the learning of each step separately. We also excluded abstracts, conference papers, posters, reviews, editorials, and opinion letters written in English, and any type of document written in a language other than English.

### 2.4. Search Strategy

We searched EMBASE, PubMed, Web of Science, Google Scholar, and CINAHL databases to ensure literature coverage [23]. We searched each database from the inception to September 30, 2019, and we used the following search strategy: (“procedural”) AND (“skill” OR “skills” OR “competence” OR “competency”) AND (“training” OR “teaching” OR “instruction” OR “assessment”). We used broad search criteria to maximize the number of potential candidate articles. In the Google Scholar database, patents, and citations were excluded.

### 2.5. Study Selection

One author screened titles and abstracts. After removing duplicates, the same author retrieved articles eligible for full-text reading. Then, two authors assessed the articles’ eligibility, resolving disagreements through discussion. Additionally, we conducted a backward search [22], i.e., we manually searched the references list of included articles for possible new eligible articles.

### 2.6. Data Collection

One author collected the data by completing a form previously tested for each included article; then, the data was verified by a second author. We classified the data extracted by specialty, procedure, instructional modality, study details (such as study design and participants), and aspects of the sequence of steps (whether the study reported that the sequence was explicitly taught or assessed, strategies reported to explicitly teach or assess the sequence, outcomes, results, validity of instruments to assess the sequence). We did not perform a meta-analysis due to the variety of study designs and outcomes found in the included articles.

### 2.7. Validity of Assessment’s Instruments

We searched evidence for validity using the practical guidelines proposed by Borgersen et al. [24]. On the **content** validity side, they propose to consult people with experience in the procedure to design or make adjustments to an instrument. Regarding **response** validity, they suggest using standardized instructions and blinded raters to minimize biases. To test **internal structure** validity, they recommend determining the reliability of scores (i.e., the instrument provides the same results each time it is used to assess residents) through statistical methods. To gather evidence regarding **relationship to other variables**, they propose to determine the correlation between assessment scores and other variables, such as experience or proficiency level. Finally, for **consequential validity**, they propose to determine the consequences that the test had, for example, defining a pass-fail score.

### 2.8. Quality Assessment

Two authors assessed the quality of the included articles independently, and resolved disagreements through discussion. We used the Medical Education Research Study Quality Instrument (MERSQI) [25,26], a rating scale to assess the methodological quality of quantitative studies in medical education research. With this instrument, it was possible to rate the study design, sampling, type of data, validity, data analysis, and outcomes of each article [25,26]. Moreover, MERSQI defines a maximum score of 3 points for each component, and rates each study with a maximum total score of 18 points.

## 3. Results

### 3.1. Study Selection

**Figure 1:**
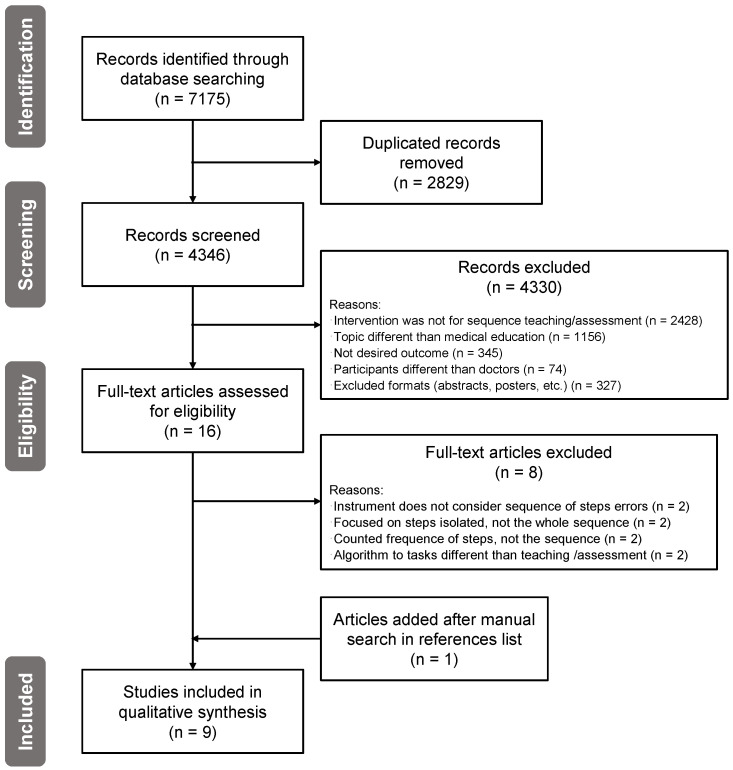
PRISMA flowchart, based on [22].

In total, we identified 7175 articles by searching the databases: 2724 from Web of Science, 2368 from EMBASE, 1928 articles from PubMed, 120 from Google Scholar, and 35 from CINAHL. 4346 articles remained after removing duplicates. As a result of screening titles and abstracts using the eligibility criteria mentioned earlier, we selected 16 articles for full-text assessment. After the full-text assessment, we selected eight articles for inclusion, and we excluded eight articles: two because the assessment instrument does not allow evaluation of whether residents made sequence errors, two because the teaching strategy focused on each step separately and not on the whole sequence, two where the authors determined the frequency of each procedural step (not the sequence), and two that used a machine learning algorithm for tasks other than teaching and assessing the sequence of steps. We included one article after the manual search in the references list of the articles fully read. The PRISMA diagram can be found in Figure 1.

### 3.2. Characteristics of Included Articles

The included articles considered procedures from emergency medicine [27,28], podiatry [29,30], general surgery [31,32], endovascular surgery [33], general medicine [34], and dentistry [35]. The educational strategies that the included studies used were video-based strategies [27,29,32,34,35], simulation [28,30,33,34,35], lectures [27,35], bedside teaching [29], and interviews [31]. Five studies included only undergraduate students as participants [29,30,32,34,35] and one study only postgraduate students [28]; two studies included both undergraduate and postgraduate students [27,31]; and one included only faculty members who were novices performing the procedure [33]. Two studies focused on collecting validity evidence for their instrument [27,31], and seven studies on testing an instructional strategy [28,29,30,32,33,34,35]. Two of the latter type of studies taught the sequence explicitly and also assessed it [28,32], two studies taught the sequence explicitly but did not assess it [29,35] and three studies assessed the sequence of steps without explicitly teaching it [30,33,34]. MERSQI median score and interquartile range for the nine studies included was median = 11.5, Q1 = 10, Q3 = 13.5. A detailed overview of the included articles is presented in the Supplementary Material.

### 3.3. Strategies to Teach the Standard Sequence of Steps

Table 1 shows the strategies found in each study. Three studies reported the use of videos to demonstrate the standard sequence of steps as a strategy [29,32,35]. In Lehmann et al. [29], they used videos with the steps logically ordered together with bedside teaching, while in Guerlain et al. [32], they used only videos repeating each step three times before moving on to the next step. Aragon and Zibrowski [35] showed a video with the step-by-step of the procedure during the class, and they gave the students a DVD with the video so they could review it whenever they wanted. Another strategy was used by Lammers [28], which consisted of informing students of the steps executed in the wrong sequence as soon as they made these mistakes while performing the procedure on a model.

### 3.4. Strategies to Assess the Learning of the Sequence of Steps

Table 2 shows the strategies found in each study. In four studies, the authors reported that the sequence of steps was assessed by asking participants to perform the procedure [27,28,30,33]. In Brenner et al. [33], the evaluators subjectively rated the participants’ performance in a virtual reality simulator. In Chapman et al. [27], the participants performed the procedure in a computer simulation scenario. Evaluators in the study conducted by Lammers [28] asked the participants to perform the procedure on a model. Similarly, in Lehmann et al. [30], evaluators assessed the participants by asking them to perform the procedure on a mannequin.

In two studies, participants were asked to say their actions aloud [27,31]. In Balayla et al. [31], they were asked to say the steps in the sequence they remembered them, while in Chapman et al. [27], they had to verbalize the steps while performing the procedure on an animal model. Two other studies asked participants to write the procedure’s steps in the proper sequence [27,34], and in another study, the participants answered three true/false and multiple-choice questions about the sequence of steps [32].

### 3.5. Instruments to Assess the Learning of the Sequence of Steps

The evaluators used different instruments with different rating scales to assess the sequence of steps. In Brenner et al. [33], they used a 5-point Likert scale. In Cheung et al. [34], they assigned 13 points to the sequence of steps written by the students (the procedure has 13 steps). In Lehmann et al. [30], they assigned a score to each step considering whether it was done in the correct position of the standard sequence: 2 points if it was done in the correct position; 1 point if it was done in the wrong position, or 0 points if it was not done. In Chapman et al. [27], they considered adherence to the standard sequence of steps as one of four items to assign a score to each step. On the other hand, some studies did not use rating scales. In Guerlain et al. [32], evaluators used true/false and multiple-choice questions about the sequence of steps. In Lammers [28], they counted the number of sequence errors that the participants committed.

### 3.6. Outcomes to Assess the Learning of the Sequence of Steps

Table 2 shows the outcomes found in each study. Six studies measured the adherence to a standard sequence of steps [27,28,30,32,33,34]. To measure this outcome, the evaluators used the instruments mentioned in the previous section. Moreover, two studies measured the number of omissions and steps done in the wrong sequence [27,31]. In Balayla et al. [31], they used this outcome to penalize the checklist’s total score with the number of omissions and steps done in the wrong sequence. On the other hand, in Chapman et al. [27], they considered both errors as one of four items to assign scores to each step.

### 3.7. Validity of Instruments to Assess the Sequence of Steps

Table 2 shows the evidence found in each article. Three studies used assessment instruments without validity evidence to assess the sequence of steps [32,33,34], i.e., experts in the procedure involved were not asked for their opinion on the instrument, nor was the consistency of these instruments evaluated when assessing residents. Three studies presented evidence for content validity since experts were asked about the instrument’s suitability: Balayla et al. (2012) [31] developed the instrument based on checklists previously designed, Chapman et al. (1994) [27] asked emergency thoracotomy experts to develop all the assessments they used, and Lehmann et al. (2015) [30] tested the instrument with student tutors and faculty. One study presented evidence for internal structure validity (specifically in the animal model and computer assessment) [27], which means that such instruments contain different questions to assess the same skill. They used Cronbach’s Alpha to determine evidence for this type of validity, and found positive values of this metric. Three studies presented evidence for response validity: Balayla et al. (2012) [31] used raters out of the resident’s training program, Lammers (2008) [28] and Lehmann et al. (2015) [30] used experts that independently rated the participants. Only one study presented evidence for consequential validity [31] deciding the approval or failure of the training course with the instrument under analysis. They used a ROC curve between novices and experts to determine the pass–fail score. Moreover, three studies presented evidence for more than one type of validity: Lehmann et al. (2015) [30] show evidence for content and response validity, Chapman et al. (1994) [27] for content and internal structure validity and Balayla et al. (2012) [31] for content, response, and consequential validity.

### 3.8. Effectiveness of Strategies to Teach the Standard Sequence of Steps

Table 1 shows the outcomes and effectiveness of the strategies found. In Guerlain et al. [32], the results showed that the students’ performance in the true/false and multiple-choice questions improved after the intervention. Lammers [28] found no significant differences between control and experimental groups when pointing out to students the sequence errors they made. In Lehmann et al. [29], they asked the students about the benefits of videos for learning and self-confidence, and the most frequent answer was that videos showed them a concrete and standardized sequence of steps. Finally, Aragon and Zibrowski (2008) [35] measured the grades of the participants in a final course test and found a positive correlation between the use of the videos and the grades, but they only found it in one of the three procedures (all-ceramic crown preparation and provisional restoration procedure). When comparing the grades, they found that the intervention participants obtained higher test scores than those of the control group, but only for the procedure mentioned.

## 4. Discussion

We searched the literature for studies reporting strategies to teach and assess the sequence of steps in procedural skills training. The results show that the teaching of a standard sequence of steps and the assessment of this aspect is rarely reported in procedural skills training studies. Regarding the quality of studies, the MERSQI median score of the nine studies included is moderate [26]. Studies’ quality does not allow us to determine whether teaching a standard sequence of steps or assessing it explicitly have a positive impact on learning this aspect. This refers to the lack of validity evidence for instruments to explicitly assess the standard sequence of steps’ learning [27,28,30], and the need to optimize teaching strategies designed for this aim [28,32,34,35].

A strategy used to teach the standard sequence of steps was simulation-based feedback. The use of simulation allows training in a safe environment without harming patients [24], it is effective as a learning modality of procedural skills [21], and in some studies has proven to be cost-effective [36]. However, the immediate feedback used by Lammers [28] did not show significant differences between both groups. Despite this result, this strategy prevents students from keeping the errors in their long-term memory, and, thus, students are less likely to commit the same errors in the future [37].

To determine the effectiveness of an instructional strategy, it is recommended to have an adequate alignment between the teaching strategy and the assessment task to evaluate the skill’s learning [38]. Two included studies presented some level of alignment between the teaching strategy reported and the assessment task, one of them had positive effects [32] while the other did not [28]. Hence, the effectiveness of strategies to teach the standard sequence of steps explicitly remains unclear.

Six studies assessed adherence to a standard sequence of steps [27,28,30,32,33,34]. They used a variety of instruments to measure this outcome (Likert scale, different scoring protocols, multiple-choice, and true/false questions). Some authors perceived that checklists do not usually allow assessing adherence to a standard sequence of steps or the omission of steps [28,30]. We support this perception because we found few studies measuring these outcomes. Therefore, further research might help to develop assessment instruments that consider the omissions and the adherence to a standard sequence of steps, thus, assessing the sequence of steps during procedural skills training with instruments suitable for this purpose. Moreover, the videos collected in training sessions could be used to identify these patterns with automated methods, such as event detection, and, therefore, to develop a sequence of steps assessment instrument.

Regarding the validity of the instruments we collected, four studies presented some type of validity [27,28,30,31], while three studies did not present evidence of validity [32,33,34]. The lack of instruments’ validity to explicitly assess the sequence of steps is a research gap to be addressed [27,28,30], which can be covered using contemporary validity frameworks, following the recommendation of Borgersen et al. [24].

Teaching a standard sequence of steps and assessing its learning is crucial to prepare residents to deal with procedural variability [6,7,13]. This incorporation will improve the residents’ training and prepare them better for future challenges [7]. Even these useful approaches in medical education can be explored in other educational areas, like internships for business students [39]. However, it has been seen that instructors struggle to guide residents on what is a principle and what is a preference, and they typically are not explicit about the procedural variability [6,40]. We propose the use of Surgical Process Models and Surgical Data Science to explicit the variability in the sequence of steps of a procedure, and also to differentiate between principles and preferences.

**Figure 2:**
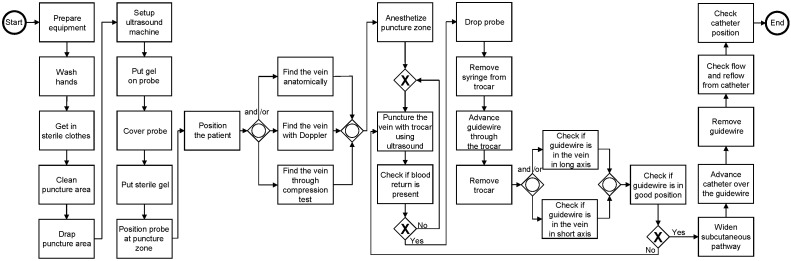
Surgical Process Model depicting the central venous catheter installation procedure, particularly the activities, decision points, and paths involved [17].

Surgical Process Models (SPM) is a recent area dedicated to modeling surgical procedures as processes [10,20]. SPM models are ‘a simplified (formal or semiformal) representation of a network of surgical or surgery-related strategies and their relationships’ [10], which enables the understanding of surgical procedures as a collection of steps that are sequentially ordered [10,20]. The formality of the modeling languages on which SPM models are built upon allows visualizing decision points along the procedure, defining what is a principle and what is a preference. Figure 2 shows a Surgical Process Model of the central venous catheter installation procedure [17], which is commonly present in anesthesiology training programs. This model explicitly states the sequence of steps to perform this procedure, the decision points where it is possible to choose between steps representing different options, and where parts of the procedure could be repeated.

As a complement to Surgical Process Models, Surgical Data Science (SDS) allows the analysis of surgical procedures through data [41]. Surgical Data Science is a field whose main goal is to extract information and insights from data obtained from surgical procedures [42]. It aims to improve healthcare quality by measuring, modeling, and quantifying improvements based on data [41]. This approach requires creating platforms to capture and curate the data, designing algorithms to use the captured data, and, thus, obtaining insights to improve surgical care [41,43].

Both Surgical Process Models [10,20] and Surgical Data Science [41] might help to report the sequence of steps in research studies and to explicitly teach or assess the order of steps in procedural skills training [10,20,41], allowing the understanding of surgical procedures as a set of steps that are sequentially ordered. Using the approaches proposed by both areas would help to close the gaps we identified in this literature review; and to make explicit the order of steps as a learning objective, producing the desired alignment between objective, teaching strategy, and assessment [38]. Specifically, Surgical Process Models and Surgical Data Science might help to report the procedure’s standard sequence of steps in research studies, to compare surgical approaches, to explicitly teach the standard sequence of steps and assess the adherence to the standard sequence in procedural skills training [10,20,41]. Furthermore, these models would help to make the sequence of steps explicit as a learning objective, producing the desired alignment between objective, teaching strategy, and assessment [38].

Instructors can use an SPM as a procedural diagram to depict the procedure’s standard sequence of steps. An SPM resembles a flowchart of the procedure that shows the steps and their standard sequence of execution [17]. Teaching a surgical procedure using an SPM might help students visualize the sequence of steps, focus the training on the sequence of steps, visualize what is a principle and what is a preference, rehearse the sequence in the parts of the procedure that were difficult to perform [44,45], and provide feedback focusing on the sequence of steps. These strategies could complement the information provided by videos or the instructor of the training.

Moreover, using Surgical Data Science techniques through data analysis supports the strategies mentioned above [46]. The data needed can be obtained from video recordings of students’ performances. Using the steps defined by a Surgical Process Model as a reference, it is possible to obtain detailed data on the order of steps that students followed from these videos [47]. Algorithms can then use the data to visualize and quantify adherence to a predefined order, identify omissions and commissions performed by students and give feedback about the order of steps based on data. In particular, the use of Process Mining [48], a discipline whose algorithms are used to analyze the adherence of the sequence of steps of processes to an expected sequence, seems to be the most appropriate family of algorithms to gain insights regarding the sequence of steps. The support of Process Mining techniques helps to allocate efforts when teaching the order of steps (e.g., knowing what stages of the procedure need to be strengthened) and assessing the order of steps based on data.

The strategies mentioned in the prior sentences are relevant because experts omit about 70% of the information that students need during their learning process [40], and it is difficult for experienced surgeons to share their mental models [6,49,50]. Additionally, these strategies would serve to simulate real situations that rarely occur and expose students to unusually performed procedures [51].

Despite the potential benefits that Surgical Process Models bring to procedural skills training, implementing and creating them is not straightforward. One reason is that Surgical Process Models need to be comprehensive, i.e., the Surgical Process Model has to represent most of the procedural variations that a surgical procedure has, thus, making the model suitable to different patients, surgeons, and hospital resources [10,12]. To address this issue, the variations that residents need to know at the end of the training (e.g., in a competency-based training) can be selected through consensus, and, thus, including them to create a suitable Surgical Process Model. A second reason is that it remains unclear whether following the sequence defined by Surgical Process Models ensures positive patient outcomes, and further research is needed to analyze the impact of following the sequence defined by a Surgical Process Model on outcomes. Furthermore, it needs to be researched how to assess the sequence of steps with the help of SPMs. Such an instrument should consider the variability that residents’ performances and the differences that the steps have, in terms of their relevance to the outcome of the procedure (e.g., omitting ‘remove guidewire’ is much more serious than omitting ‘cover probe’ in the procedure shown in Figure 2).

Regarding limitations on implementing Surgical Data Science, it might be challenging to collect the data needed to analyze surgical procedures from a data science point of view, due to the time and resources required to generate them. Another reason is that it is necessary to carefully choose the most appropriate analysis technique among those Surgical Data Science offers, and it should provide a sufficient level of reliability and simplicity to increase the likelihood of adoption of these developments. Finally, we acknowledge that procedural training includes other aspects beyond the sequence of steps [19,39]. Therefore, the inclusion of the sequence of steps should interact with current successful strategies to teach the other aspects to avoid hindering the training.

A limitation of this study is that we did not analyze the effectiveness of the teaching strategies described through meta-analysis since they all used different ways of measuring it and the misalignment found between teaching and assessment strategies. Another limitation is that the effectiveness of the strategies described to teach the sequence of steps could be biased since most of the studies found did not have the sequence of steps as the unique focus.

## 5. Conclusions

The standard sequence of steps is an aspect rarely reported in procedural skills training studies. The included studies presented high variability in the strategies and instruments used to explicitly teach and assess the learning of a standard sequence of steps. Moreover, the studies’ quality prevents determining whether the strategies for teaching or assessing the sequence of steps have a positive impact on the learning of this aspect. Therefore, more research is needed to find methods and strategies that ensure the learning of this aspect during procedural training and, consequently, prepare residents to deal with procedural variability better. Using innovations such as Surgical Process Models and Surgical Data Science might enable the design of new strategies and instruments to incorporate the standard sequence of steps in procedural skills training.

## Figures and Tables

**Table 1:** Teaching the standard sequence of steps: strategies, outcomes and effectiveness.

Authors	Instructional Strategy	Outcome	Effectiveness
Aragon and Zibrowski [35]	Video demonstration	Grades obtained in the practical exam (evaluators rate students using a twenty-eight items instrument).	Effective only for one of the three procedures analyzed. *
Guerlain et al. [32]	Video demonstration	Adherence to the standard sequence of steps (participants answered questions about the sequence of steps).	Effective, it improved performance in procedural questions. *
Lammers [28]	Informing subjects of all performance and sequence errors immediately	Adherence to the standard sequence of steps (evaluators counted the number of sequence errors).	There were no significant differences between the control and experimental group.
Lehmann et al. [29]	Video demonstration	Benefits for learning (participants answered an open question).	Videos showed a concrete and standard sequence of steps.

^*^ Statements of Aragon and Zibrowski [35] and Guerlain et al. [32] were tested using the corresponding statistic test.

**Table 2:** Assessing the learning of the sequence of steps: strategy, outcomes, and validity of instruments.

Authors	Strategy	Outcome	Validity of Instrument
Balayla et al. [31]	Participants say the steps aloud.	Omissions (evaluators counted these errors and discounted them from the checklist’s total score).	Content, response, and consequential validity.
Brenner et al. [33]	Performing the procedure in a virtual simulator.	Adherence to the standard sequence of steps (evaluators used a 5-point Likert scale).	None.
Chapman et al. [27]	· Students write the steps. · Performing the procedure in a computer simulation. · Performing the procedure on an animal model, and saying the steps aloud.	Adherence to the standard sequence of steps and omissions (both outcomes were evaluated by assigning a score to each step using a rating scale).	Content validity for all assessment strategies. Internal structure validity for animal and computer assessment.
Cheung et al. [34]	Participants write the steps in the proper sequence.	Adherence to the standard sequence of steps (evaluators assigned points to each participant).	None.
Guerlain et al. [32]	Students answer a test with questions about the sequence aspect.	Adherence to the standard sequence of steps.	None.
Lammers [28]	Performing the procedure on a model.	Adherence to the standard sequence of steps (evaluators counted the number of sequence errors).	Response validity.
Lehmann et al. [30]	Performing the procedure on a mannequin.	Adherence to the standard sequence of steps (evaluators rated each step through a rating scale, considering whether the step was omitted, done in the correct or incorrect position).	Content and response validity.

## Data Availability

Not applicable.

## Supplementary Materials

A detailed overview of the included articles, the data collection form and the review protocol can be downloaded at: https://www.mdpi.com/article/10.3390/ijerph20021436/s1.

## Abbreviations

The following abbreviations are used in this manuscript:

BPMN	Business Process Model and Notation
PRISMA	Preferred Reporting Items for Systematic Reviews and Meta-Analyses statement
EMBASE	Excerpta Medica dataBASE
CINAHL	Cumulative Index to Nursing and Allied Health Literature
MERSQI	Medical Education Research Study Quality Instrument
SPM	Surgical Process Model
