# Supplementary material for: The Sequence of Steps: A Key Concept Missing in Surgical Training—A Systematic Review and Recommendations to Include It
Source: Int J Environ Res Public Health. 2023 Jan 12;20(2):1436. doi: 10.3390/ijerph20021436 (PMC9859547; doi:10.3390/ijerph20021436)
Supplement: Supplementary file 1 [file ijerph-20-01436-s001.zip › ijerph-2065357-supplementary.pdf]

Supplementary material: Overview of included articles.

Authors	Specialty	Procedure	Instructional Modality	Study Design	Participants	MERSQI
Aragon and Zibrowski [35]	Dentistry	All-ceramic crown preparation.<br>Full gold crown preparation.<br>Posterior porcelain-fused-to-metal fixed partial denture.<br>*(the 3 procedures with provisional restoration)	Lectures, simulation on mannequin, videos	Case-control design	55 undergraduate students	9.5
Balayla et al. [31]	General surgery	Inguinal hernia repair with mesh in men.<br>Laparoscopic cholecystectomy.<br>Open right hemicolectomy.	Interview to validate the assessment instrument.	Cross-sectional	35 subjects (‘Novice’ group: undergraduate medical students, residents in first and second postgraduate year (PGY1-2). ‘Expert’ group: residents in third to fifth postgraduate year (PGY3-5) and program staff.)	11.5
Brenner et al. [33]	Endovascular surgery	Resuscitative Endovascular Balloon Occlusion of the Aorta (REBOA)	Virtual Reality Simulation (VRS)	Pre-post test	13 faculty members who are novice interventionalists.	10
Chapman et al. [27]	Emergency Medicine	Opening the chest.<br>Pericardiotomy.<br>Aortic cross-clamping.	Lectures, videos	Sequential post-test	18 subjects (6 undergraduate medical students, 6 fourth postgraduate year (PGY4), 6 faculty)	14

Cheung et al. [34]	General Medicine	Lumber Puncture	Simulation in part-task simulator, videos	Pre-post test	29 undergraduate pre-clerkship medical students (14 control, 15 intervention)	15.5
Guerlain et al. [32]	General Surgery	Laparoscopic Cholecystectomy	Videos	Pre-post test	30 undergraduate medicine students (14 control, 16 intervention)	12.5
Lammers [28]	Emergency Medicine	Posterior epistaxis management	Simulation in a model	Prospective, repeated-measures	13 first postgraduate year (PGY-1) residents, 13 second postgraduate year (PGY-2) residents, and 2 third postgraduate year (PGY-3) residents (15 control, 13 intervention)	10.5
Lehmann et al. [30]	Pediatrics	Pediatric Basic Life Support (PBLS)	Simulation on a mannequin	Randomized trial	57 undergraduate medical students (30 control, 27 intervention)	13.5
Lehmann et al. [29]	Pediatrics	Physical examination procedures	Videos, bedside teaching	Survey after intervention	175 undergraduate medical students	9.5

PRISMA guidelines		
3	Rationale	Relevance of procedural skills<br>Procedural competence has typically been assessed with checklists and global scales.<br>These methods downplay the importance of decomposing the procedure into discrete components for step-by-step training and objective assessment.<br>There are literature showing that the previous ideas can be effective for procedural skills training.<br>Also, there is recent interest in the development of step-by-step methodologies for procedural skills development.<br>To date, there is no review describing how the order of steps of a surgical procedure has been incorporated into training and assessment of procedural skills.
4	Objectives	Determine how the order of steps of a surgical procedure have been incorporated in procedural skills training.
	Research Questions	How has the order of steps of a surgical procedure been incorporated in procedural skills teaching and training?
6	Elegibility criteria	P — Population — doctors (instructors or students) who perform a procedure for training and/or assessment purposes.<br>I — Intervention — Any with the aim of training and/or assessment of procedural skills.<br>C — Comparison — Those who don't participate of the intervention (control v/s intervention group), in case of evaluation of interventions. If it is a design development, the current literature.<br>O — Outcomes — Formalization/representation of the procedure; performance analysis of the procedure; development, validation and/or evaluation of tools or instruments. Articles should consider any way to explicitlyteach and/or assess the sequence of steps.<br>S — Study design — The ones that allow to get the outcomes described before.<br>Articles in english. All the articles other than systematic review, abstracts and books, from inception to September 30, 2019.
7	Information sources	Databases:<br>PubMed 1928<br>EMBASE 2368<br>CINAHL 35<br>Web of Science 2724<br>Google Scholar 120
8	Search string	("procedural") AND ("skill" OR "skills" OR "competence" OR "competency") AND ("training" OR "teaching" OR "instruction" OR "assessment")
	Date	September 30, 2019
	PubMed	(((("procedural"[Title/Abstract]) AND ("skill"[Title/Abstract] OR "skills"[Title/Abstract] OR "competence"[Title/Abstract] OR "competency"[Title/Abstract])) AND ("training" [Title/Abstract] OR "teaching"[Title/Abstract] OR "instruction"[Title/Abstract] OR "assessment"[Title/Abstract]))) AND "english"[Language] . LANGUAGE: ENGLISH
	EMBASE	procedural':ab,ti AND ('skill':ab,ti OR 'skills':ab,ti OR 'competence':ab,ti OR 'competency':ab,ti) AND ('training':ab,ti OR 'teaching':ab,ti OR 'instruction':ab,ti OR 'assessment':ti) AND [english]/lim AND [embase]/lim. LANGUAGE: ENGLISH
	CINAHL	AB "procedural" AND AB ("skill" OR "skills" OR "competence" OR "competency") AND AB ("training" OR "teaching" OR "instruction" OR "assessment") AND TI "procedural" AND TI ("skill" OR "skills" OR "competence" OR "competency") AND TI ("training" OR "teaching" OR "instruction" OR "assessment"). LANGUAGE: ENGLISH
	WoS	(TS=(("procedural") AND ("skill" OR "skills" OR "competence" OR "competency") AND ("training" OR "teaching" OR "instruction" OR "assessment"))) *AND* LANGUAGE:(English)
	Google Scholar	allintitle: ("procedural") AND ("skill" OR "skills" OR "competence" OR "competency") AND ("training" OR "teaching" OR "instruction" OR "assessment"). LANGUAGE: NO RESTRICTION
9	Study selection	Screening will be made based on title and abstract. Then eligibility criteria will be applied.
10	Data collection process	Data collection form. It will be verified with a random selection of primary studies and comparing the content of the form filled by both reviewers.
11	Data items	Those defined in the data collection form.
12	Risk of bias in individual studies	-
15	Risk of bias across studies	-

**Basic information**	Title
	Year of publication
	Author(s)
	Journal
	Authors workplace
	Data extractor
	Data checker

**General content**	Speciality
	Procedure analyzed
	Objectives
	General purpose: teaching or assessment?
	Learning strategy
	Study design
	Do the article contain the steps of the procedure?

**Materials**	Population included
	Population excluded
	Type of population (instructors/students)
	Source of data

**Methods**	Outcomes
	Experiment design

**Results**

**Research gaps - Future trends**

**Comments**
